# Supplementary material for: Crop diversity induces trade-offs in microbial biopesticide susceptibility that could delay pest resistance evolution
Source: PLoS Pathog. 2025 May 20;21(5):e1013150. doi: 10.1371/journal.ppat.1013150 (PMC12091894; doi:10.1371/journal.ppat.1013150)
Supplement: S1 Table — (DOCX) [file ppat.1013150.s001.docx]

Table S1 | **Impact of crop diet and fungal pathogen on larval survival: GLMM estimates.** Parameter estimates from a generalised linear mixed model (with logit link) of larval survival as a function of food plant and fungal isolate treatment. The interaction p-value was estimated using parametric bootstrapping to compare models with and without the interaction term. Note that the residual variance for a binomial model is fixed at 1.

| term | estimate | | Std. error | z-statistic | pb P-value |
| --- | --- | --- | --- | --- | --- |
| (Intercept) | | -1.802 | 0.162 | -11.137 |  |
| Crop (Maize) | | 1.802 | 0.155 | 11.661 |  |
| Crop (Tomato) | | 1.856 | 0.166 | 11.165 |  |
| Isolate (*Beauveria*) | | 1.423 | 0.152 | 9.353 |  |
| Isolate (*Metarhizium*) | | 1.004 | 0.155 | 6.462 |  |
| Crop (Maize): Isolate (*Beauveria*) | | -0.607 | 0.203 | -2.991 | 0.003 |
| Crop (Tomato): Isolate (*Beauveria*) | | -0.962 | 0.217 | -4.438 |  |
| Crop (Maize): Isolate (*Metarhizium*) | | -0.621 | 0.204 | -3.043 |  |
| Crop (Tomato): Isolate (*Metarhizium*) | | -0.476 | 0.219 | -2.171 |  |
| Maternal random sd | | 0.517 |  |  |  |
| Paternal random sd | | 0.463 |  |  |  |
