# Supplementary figures and images for: Crop diversity induces trade-offs in microbial biopesticide susceptibility that could delay pest resistance evolution
Source: PLoS Pathog. 2025 May 20;21(5):e1013150. doi: 10.1371/journal.ppat.1013150 (PMC12091894; doi:10.1371/journal.ppat.1013150)

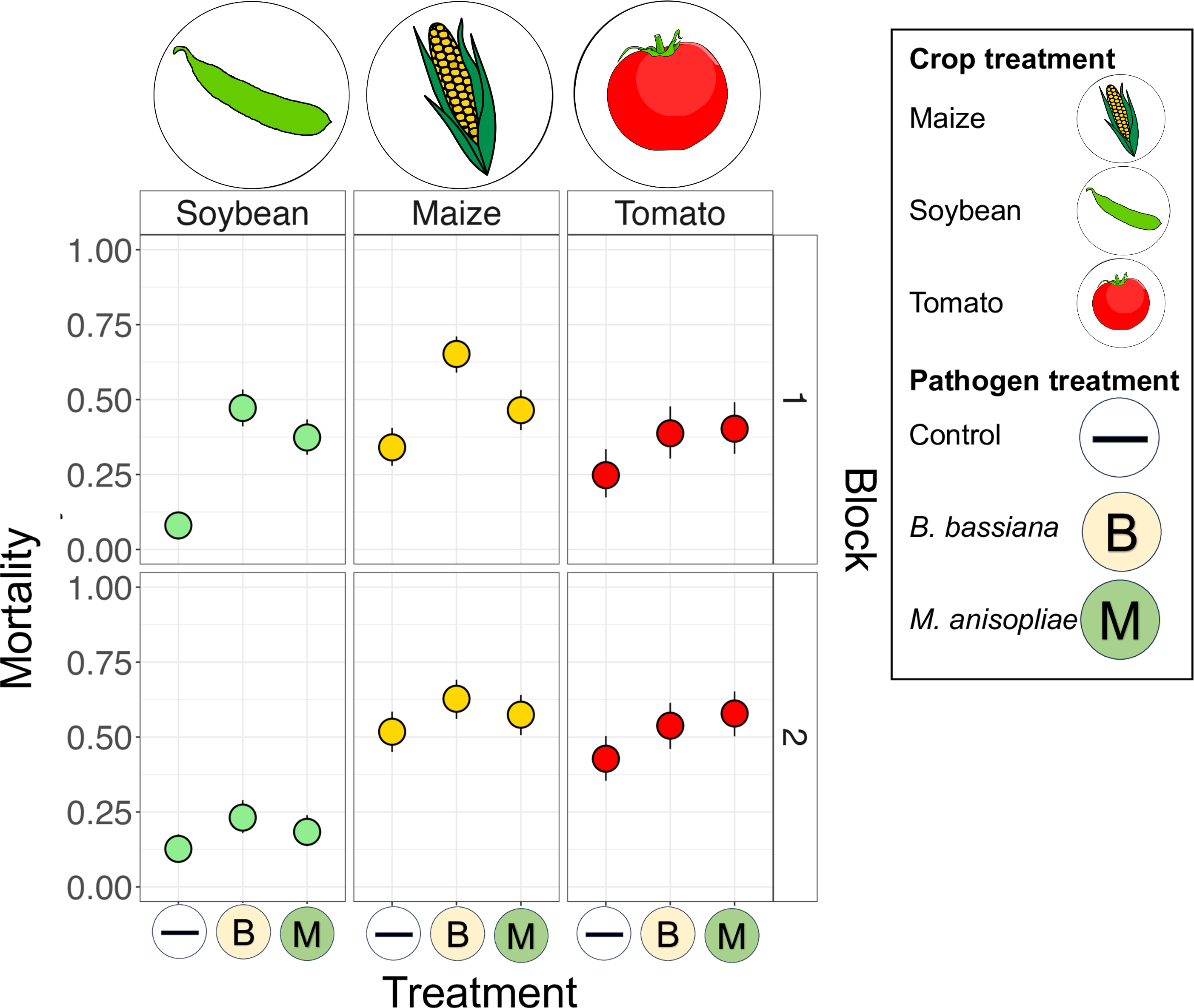

Supplement: S1 Fig — (TIF) [file ppat.1013150.s002.tif]

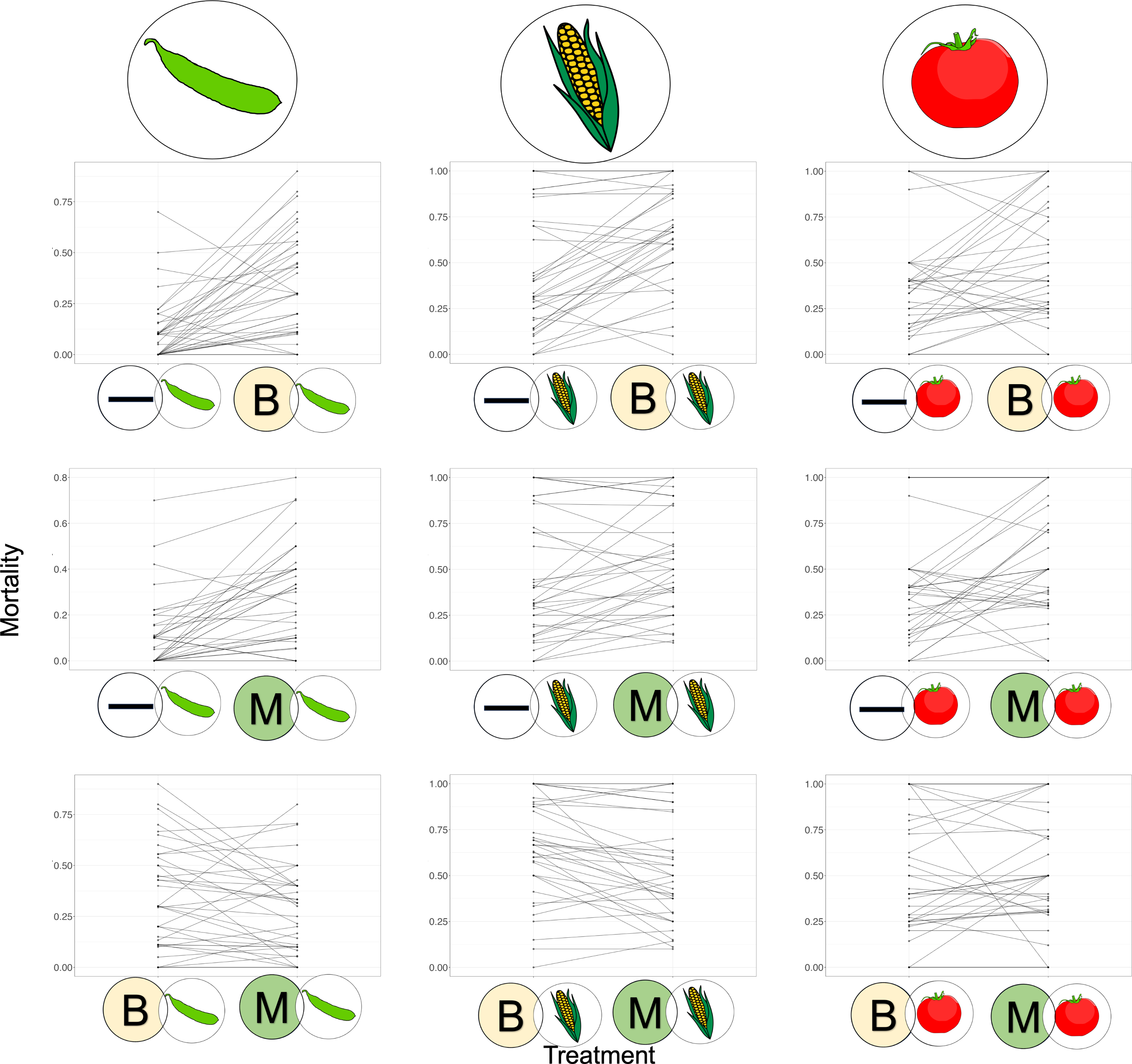

Supplement: S2 Fig — (TIF) [file ppat.1013150.s003.tif]

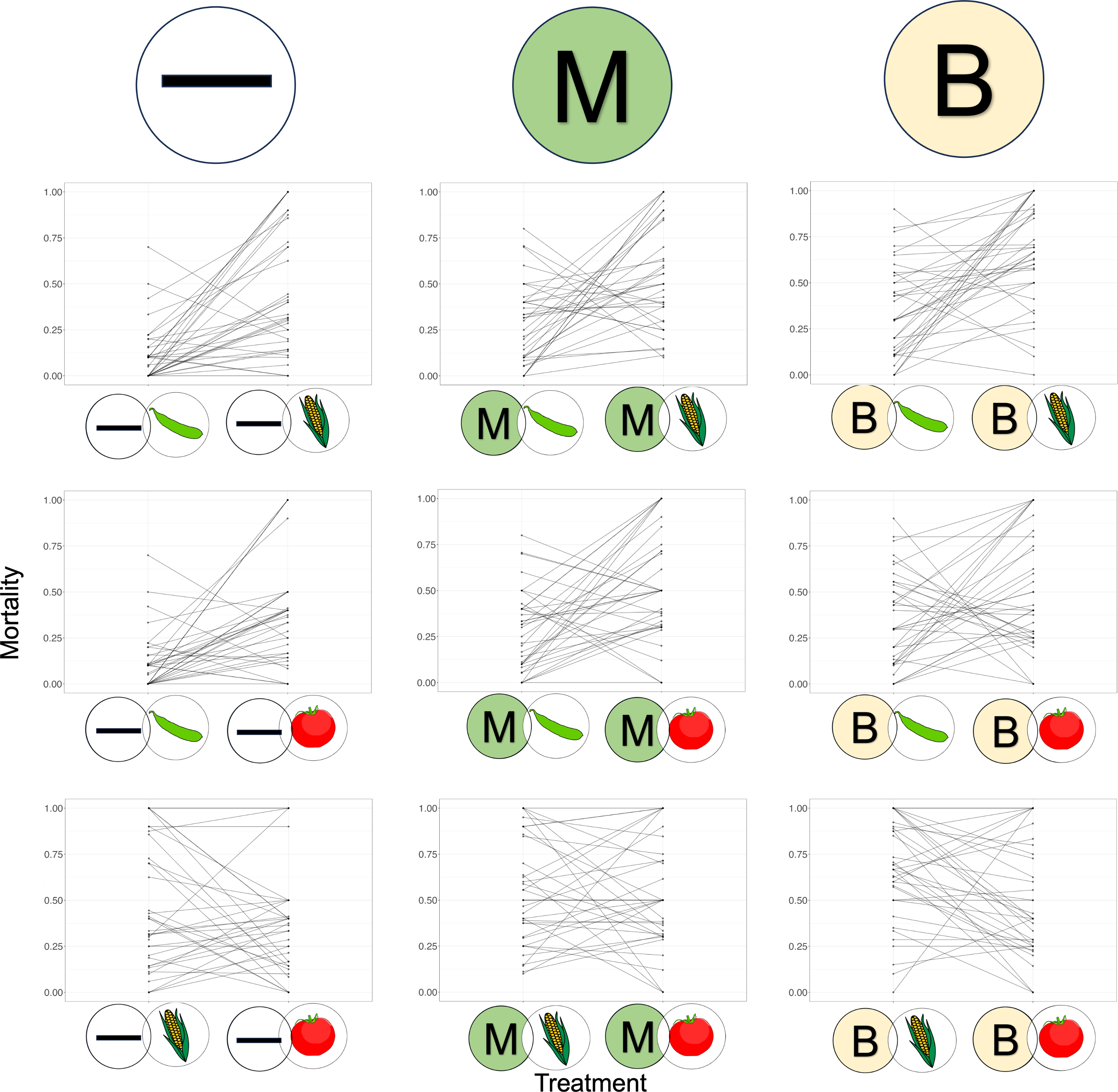

Supplement: S3 Fig — (TIF) [file ppat.1013150.s004.tif]

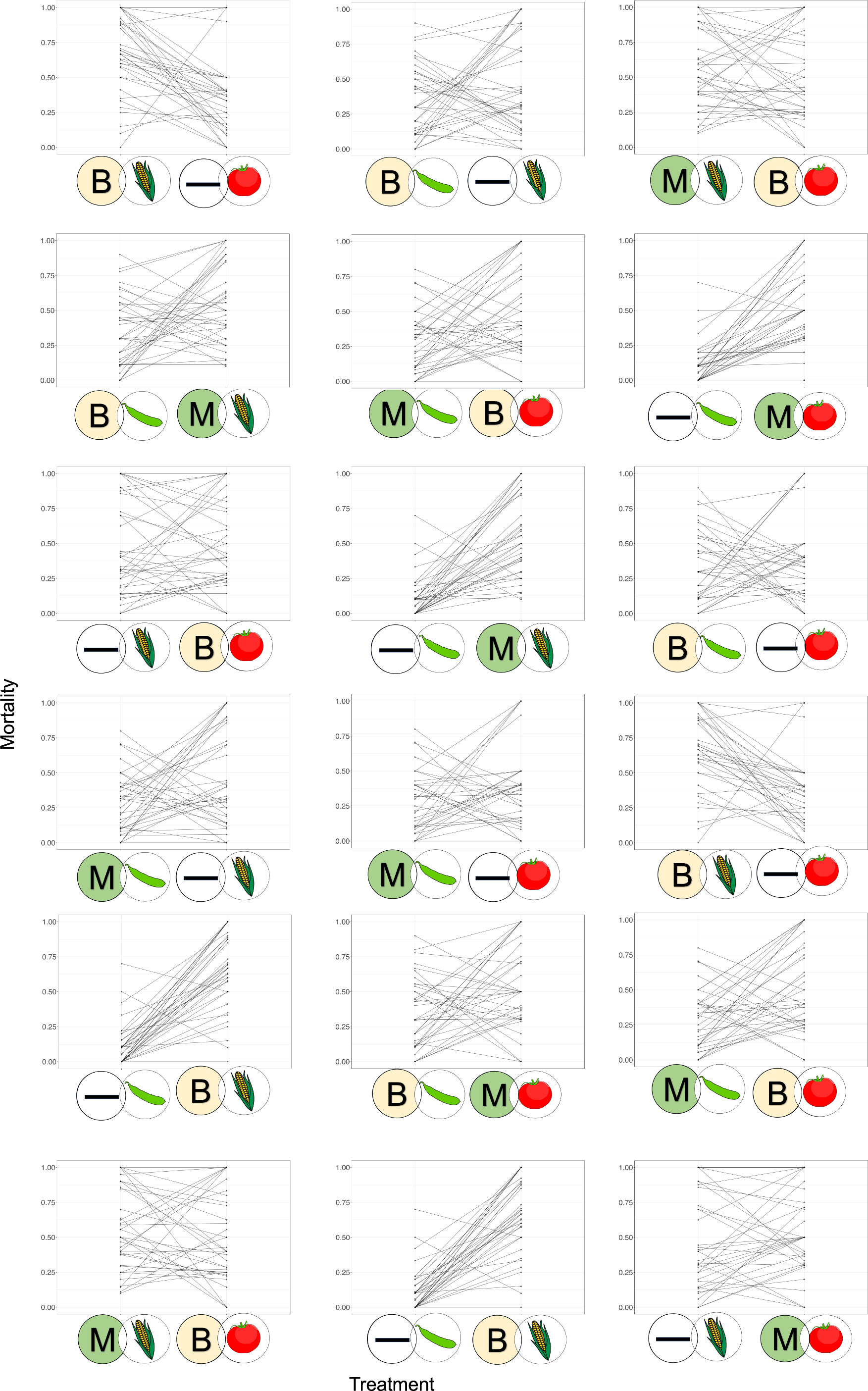

Supplement: S4 Fig — (TIF) [file ppat.1013150.s005.tif]

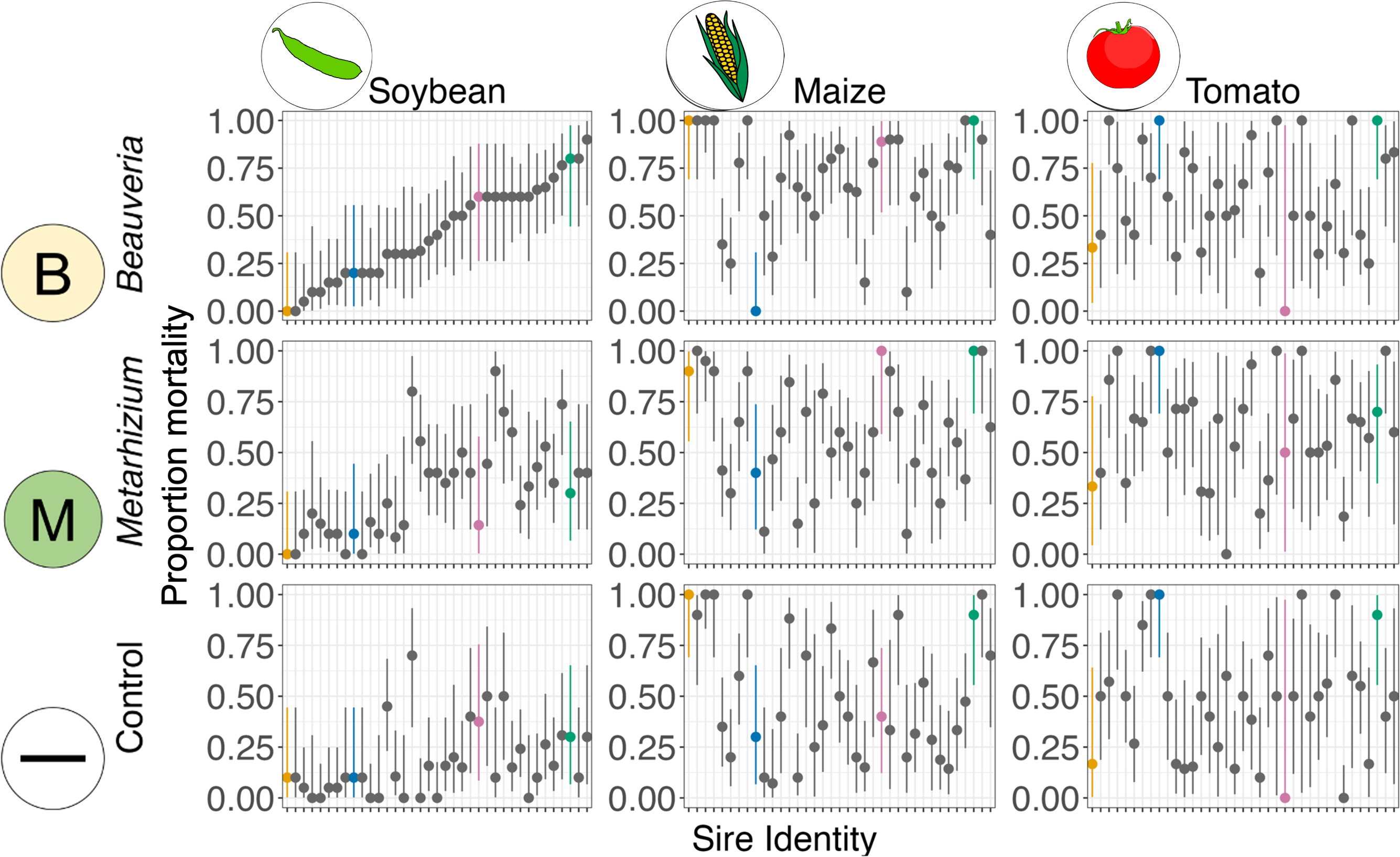

Supplement: S5 Fig — (TIF) [file ppat.1013150.s006.tif]
