# Supplementary material for: Crop diversity induces trade-offs in microbial biopesticide susceptibility that could delay pest resistance evolution
Source: PLoS Pathog. 2025 May 20;21(5):e1013150. doi: 10.1371/journal.ppat.1013150 (PMC12091894; doi:10.1371/journal.ppat.1013150)
Supplement: Attachment — Submitted filename: Manganetal_ResponsetoReviewers_PLOSPathogens.docx [file ppat.1013150.s008.docx]

**Response to Reviewers**

**Reviewers' Comments:**

Reviewer's Responses to Questions

**Part I - Summary**
Please use this section to discuss strengths/weaknesses of study, novelty/significance, general execution and scholarship.

Reviewer #1: Please see Part III.

Reviewer #2: Mangan et al. investigates timely and important topic how resistance to biocontrol agents evolves in multi host cropping. They approached topic from both theoretical and applied perspective and their experimental design was considerably large (3800 larvae) with three host species and two biocontrol agents. The experimental design seems appropriate and analysis rigorous. They find heritable variation in larvae ability to survive biopesticides. The main novelty in the manuscript is that the authors test whether host species and biopesticide identity can alter that variation. They find that there is only lesser extent of variation caused by biopesticide identity, but host species alters selection. They discuss theoretical and crop protection implications for their results. The manuscript is generally well written, and I do not have any major criticism.

Response: Thank you.

Reviewer #3: 
I really enjoyed reading this MS- it was exceptionally well written and addresses an interesting and important aspect of resistance to biocontrol agents and the genetics of pathogen susceptibility.

Strengths
The major novel aspect of this study was the size and ambition of the quantitative genetic analysis of pathogen susceptibility. Assessing even simple life history data across three host plants and two pathogens is a major undertaking and the authors should be congratulated on achieving this.
Response: Thank you.

Weakness
A few issues stood out. First was the authors' very casual use of the term 'resistance'. This can be a loaded term and to many in pest management it implies that a control strategy has been launched and then subsequently has failed due to some recent genetic change. Without doubt the authors have data on variation in pathogen susceptibility but it is a stretch to describe this as 'resistance'. I would prefer it if the authors refer to their data study as variation in susceptibility and I suspect many in the field would agree with me.

Response: We agree with this comment and recognise the challenges caused by our use of the term resistance. We recognise that we had used this word in two different contexts. Therefore, we have modified our terminology and added a small new section of text in the introduction to outline the challenges with this word (Lines 74-81). We have minimised the use of the word resistance in the context of host defence against infection to avoid confusion.

This distinction is important genetically. For example, I suspect that it's no coincidence that pathogen mortality varies from 0-100%- the authors have perhaps chosen a dose that gives the most information.
This is important as in biocontrol doses are generally chosen to give as little variability as possible. Importantly, as doses increase we would expect fewer and fewer loci to contribute to variation in susceptibility - this is an important (and perhaps unavoidable) limitation of the study and should be discussed.

Response: Thanks for this comment. We have added some lines to the text in the discussion (Lines 355- 365) to better explain our choice of doses, which as suggested was born of a wish to expose as much genetic variation as possible. However, we defend this choice as being relatively ‘field realistic’ because a recent meta-analysis (which we now cite in our revision) shows that on-farm mortality rates for biocontrol are often similar to those in our lab study. We now acknowledge that genetic variation in the field might depend on dosing regimes, which we think is an important empirical question for future study.

The authors mention polygenic resistance for pathogens- but I am not sure how well this is borne out in the field - Bacillus thuringiensis resitance is typically monogenic (references too numerous to mention); resistance to Cydia pomonella granulosis virus (the other classic example of field resistance to a pathogen biocontrol agent) is also apparently monogenic <https://doi.org/10.3390/v9090250>. Here, weak resistance in laboratory selection is often misleading but even so earlier virus work found monogenic resistance <https://doi.org/10.1016/0022-2011(82)90013-1>. Perhaps fungi are special here but this should have a more specific discussion.

Response: Reviewer 2 (here) and Reviewer 1 (in minor comments section below) have both raised issues regarding our viewpoint on the genomic complexity of resistance against pest control agents. Reviewer 2 (above) suggests that resistance to pathogens is typically monogenic. Reviewer 1 (below) suggests that resistance to chemical pesticides is often polygenic, and that some instances of resistance to pathogen-based biopesticides can be monogenic.

We recognise that this is a complex and nuanced topic; individual case studies provide contrasting evidence, and for many cases of resistance robust full-genome scans to identify all the gene loci underpinning the trait are lacking. We stand by our claim that (on average), the number of gene loci underpinning resistance to pathogens is generally greater than for chemical control agents. Note that we do not argue that resistance to either pathogens or chemical insecticides is always monogenic: merely that resistance to pathogens tends to involve more loci than resistance to chemicals*.* We have previously made this argument in detail in an Opinion article that we published in Trends in Ecology and Evolution (Mangan et al., 2023) in which we illustrated the point using a figure (Box 1: Figure 1) and cited a range of supporting literature (Cogni et al., 2016; Ffrench-Constant, 2013; Ignoffo et al., 1985; Reichelderfer & Benton, 1974; Roberts et al., 2020; Shahrestani et al., 2021; Wang et al., 2017). Recently, Legan et al. (2024) used genomic data to show that even for a single Bt crystal, there was dramatic diversity across field populations in the number and identity of genes controlling resistance phenotypes. To our knowledge, no published meta-analysis formally assesses this question quantitatively. A full response to these referee comments would be very long (perhaps a whole paper). We have written a substantial piece of text to explain our point of view and to explain that some of the case studies and papers that the referees have highlighted in their reviews actually support our position. We have appended this text at the bottom of this response letter (See: **On the variation in genomic complexity between resistance to chemical control agents and resistance to pathogens**).

In response to the reviewer’s comments, we have altered the text in our introduction (Lines 102 – 110), clarified our wording and added additional references. We have cited Legan et al. (2024), Roberts et al. (2020), Shahrestani et al. (2021), and (Wang et al., 2017) which all show that the genetic control of pathogen susceptibility variation can involve many loci. We think these changes are a welcome improvement to our paper.

The second major issue is that I feel the authors have neglected to cite and discuss a significant body of work on pathogen resistance - most of this relates to work on resistance to Bacillus thuringiensis, but there is also a wider and perhaps pertinent literature on how host plants affect susceptibility to virus (NPVs, GVs) that the authors might want to consider.
For instance, one suggestion the authors make (Lines 360-365)- applying different pathogens to different refugia as a means of resistance management - this has actually been tested experimentally (doi: 10.1111/j.1365-2664.2007.01285.x) - see also work on Bt resistance and susceptibility to nematodes (doi: 10.1111/j.1365-2664.2008.01457.x). These results challenge the generality of the concluding paragraph here ie the expection for negative trade-offs between pathogens. Importantly, previous studies have used very different pathogens/parasites and not two Entomophoralean fungi with a similar mode of action.

Note also research on fitness costs to Bt In terms of NCCRs between resistance and performance on different plants has quite a substantial literature. For example, within crop variation in Brassicas is such that it is possible to find varieties that are nearly lethal to Bt resistant genotypes doi:10.1038/hdy.2010.65- that study also showed that lower quality plants increased fitness costs of resistance across multiple insect genotypes. see also Janmaat AF, Myers JH (2005). The cost of resistance to Bacillus thuringiensis varies with the host plant of Trichoplusia ni. Proc R Soc Lond Ser B-Biol Sci 272: 1031–1038. Those two aforementioned studies found that resistant insects tended to do worse on plants of lower quality- something the authors seem not to have repeated- that is an important topic for the discussion in itself.

Response: Thank you for highlighting the relevance of studies on the fitness costs of resistance influenced by host plant variability. It's important to clarify that our study primarily investigates the sequential application of different pathogens in heterogeneous environments as a resistance management strategy, rather than the simultaneous exposure discussed in some of the suggested studies (which results in co-exposure of individuals). Nevertheless, we appreciate that the studies mentioned by the referee clarify how these dynamics influence the efficacy of resistance management strategies, particularly lower quality host plants and their effect on Bt-resistant insects. We now cite the following papers in the introduction. Raymond et al. (2007) explored integrating nucleopolyhedrovirus with Bt for pest management, illustrating that co-infection of pathogens can enhance fitness costs of Bt resistance. This concept is extended by Gassmann et al. (2009), who demonstrated how entomopathogenic nematodes synergize with Bt crops to potentially delay resistance. Janmaat and Myers (2005) contributed by showing the variability of resistance costs across different host plants, further complicating resistance management. Lastly, Raymond et al. (2011) discussed the interplay between genetic background and host plant quality, emphasizing its impact on resistance strategies. Collectively, these studies underscore the importance of considering ecological and genetic factors in managing pest resistance, highlighting integrated approaches to prolong the effectiveness of Bt crops and other biological control agents. We are pleased to highlight them in our manuscript (Lines 122- 124).

Minor comments
NCCR- Agree that its rare but also see classic example from Blackfly that was incorporated in resistance management scheme : Kurtak, D., Meyer, R., Orcran, M., & Tele, B. (1987). Management of insecticide resistance in the control of Simulium damnosum complex Medical and Veterinary Entomology, 1, 137–146.

Response: Thank you for your comment. We have acknowledged the work by Kurtak et al. (1987) and added a note that the lack of pesticide resistance trade-offs is another important reason why NCCR has not been used as widely in agriculture (Lines 88-90).

**Part II – Major Issues: Key Experiments Required for Acceptance**
Please use this section to detail the key new experiments or modifications of existing experiments that should be absolutely required to validate study conclusions.

Generally, there should be no more than 3 such required experiments or major modifications for a "Major Revision" recommendation. If more than 3 experiments are necessary to validate the study conclusions, then you are encouraged to recommend "Reject".

Reviewer #1: N/A

Reviewer #2: (No Response)

Reviewer #3: na

**Part III – Minor Issues: Editorial and Data Presentation Modifications**
Please use this section for editorial suggestions as well as relatively minor modifications of existing data that would enhance clarity.

Reviewer #1: This excellent paper reports rigorous experiments showing genetic tradeoffs across crop plants affecting the ability of a major pest to resist two fungal pathogens. The results have important implications for fundamental understanding of evolution of pathogen-host interactions and enhancing sustainability of crop protection. I have only minor suggestions for improvement: Please address the classic tactic of using non-host plants in rotations or spatial mosaics with host plants to suppress pests, including pros and cons versus using only host plants of pests as evaluated here.

Response: Thank you for this suggestion. We have added a section to address this point in the discussion in which we highlight other strategies for agricultural diversification (Lines 467-470).

This paper is about pathogens. Accordingly, for clarity and simplicity, please use the
term pathogen throughout rather than alternating between parasite and pathogen. If
there is a specific reason to consider parasites that are not pathogens in some places, explain that explicitly.

Response: We have reviewed the manuscript to ensure that the term "pathogen" is used consistently throughout. We have replaced instances of "parasite" with "pathogen" unless there is a specific reason to differentiate between the two (for example, there is an instance (Line 482) where parasite is appropriate in the context of the relationship with the pest).

Several terms in the title are ambiguous in the absence of context. Suggested revised title to improve clarity: Delaying resistance to biopesticides by exploiting trade-offs across crops in pest susceptibility to pathogens.

Response: Thank you for the suggestion to revise the title to clarify the goal, methodological approach, and the intended outcomes. We have provided a revised title that acknowledges these requests. The new title is:

*Crop diversity induces trade-offs in microbial biopesticide susceptibility that could delay pest resistance evolution*

Be clear in the introduction, abstract, and elsewhere that the paper addresses variation in the pest’s susceptibility (or resistance) to fungal pathogens. This isn’t obvious from the term “pathogen defence.”

Response: Thank you for this comment. We have revised our terminology throughout with the addition of a new section (Lines 74-81) to clarify differences in the contextual uses of ‘resistance’. We have also clarified the use of ‘pathogen defence’ to largely use the phrase ‘defence against pathogens’ instead.

L39 & 41 Revise to “genetic variation for resistance to pathogens.”

Response: We accept the referee’s criticism, but we have challenges with terminology here due to the conflict (raised by another reviewer) between different uses of the word ‘resistance’. We have revised this to ‘genetic variation for susceptibility to pathogens’.

L53. Revise to “such as biopesticides containing live pathogens.”

Response: Thank you, a revision has been made.

Cite a more apt reference: Microbial biopesticides in agroecosystems. Agronomy 2018, 8, 235; doi:10.3390/agronomy8110235

Response: Thank you for this suggestion, the reference has been added (Line 70).

L75 “Defence against microbes is typically more genetically complex than for synthetic insecticides or genetically modified crops[8, 21, 22].” Cite one or more current, rigorous references to support this claim or omit it. This claim does not seem essential here and actually detracts from the paper’s main thrust. I could not find credible, rigorous evidence in the references cited: 8, 21 or 22 (or 4). One systematic review to consider:X.-P. Lu, L. Xu, J.-J. Wang, Mode of inheritance for pesticide resistance, importance and prevalence: A review. Pestic. Biochem. Physiol. 202, 105964 (2024). Contrary to the claim above, this review reports ca. 60% of the 187 cases of lab- and field-selected pesticide resistance (mostly to synthetic insecticides) reviewed were “polygenic.” Also, 3 of the 4 cases of resistance to microbes (Bt or Bacillus sphaericus) reviewed were “monogenic.” I think negative cross-resistance has not been widely applied because it is rare. No need to invoke genetic complexity to explain this.

Response: Thank you for your comments and for drawing our attention to this reference which we now cite several times to support our arguments. We now have underlined the fact that cross-resistance is rare (Lines 90-96). We have also revised our paragraph to add citations, explain our logic and clarify the nature of the evidence for it.

See elsewhere for our response to comments about the genetic complexity of resistance.

As noted in the Discussion, the references cited for genetic complexity in the
introduction do not support the claim of defense trade-offs. So, if there is evidence that resistance to pathogens is typically complex, the inference that such complexity
promotes tradeoffs is not supported by the references cited. So, either provide clear
support for the claim that genetic complexity promotes trade-offs or omit the claim.

Response: The link between complexity and trade-offs emerges from the following logic: the greater the number of genes underlying a trait, the more likely that some of these loci will have environmentally-dependent fitness. Most studies of the genetic control of defence are conducted in near-uniform conditions, which makes it difficult to expose these dependencies, but recent work clarifies their prevalence when experiments include environmental variables and deep genomic sequencing. For example, Roberts et al. (2020) demonstrates that there are many genes underlying viral defence in a lepidopteran pest and that trade-offs involving immune defence are sensitive to nutritional resources. Of course, it is also possible to have strong pathogen defence genetic tradeoffs when the genetic basis of resistance is simple. Indeed the ‘poster child’ of host-parasite coevolution: the *Daphnia magna – Pasteuria ramosa* system is famous for the simple genetic basis of infection susceptibility with a matching-allele model and very strong genetic specificity of host susceptibility alleles. However, this does not detract from our reasoning that as the number of loci increases so does the likelihood that tradeoffs will emerge.

The evidence cited from D. melanogaster shows a positive correlation in defense across bacteria and fungi, so why is it surprising that a negative correlation was not seen between two fungal pathogens? Before publication of the D. melanogaster papers in 2017 and 2023, it might have been reasonable to expect tradeoffs between fungal pathogens. But this is no longer true and the paper should recognize this evidence and reflect the appropriate change in perspective throughout.

Response: Here we suspect that different expectations may derive from different subdisciplines, and so have revised our text to indicate that there is a widespread expectation among evolutionary ecologists that trade-offs in host resistance to different pathogen species/genotypes help explain genetic variation for pathogen susceptibility (Lines 566-571).

L413. To avoid overstatement, change “answered” to “addressed.”

Response: Thank you, this correction has been made.

L433. “However, our data call into question the prevailing expectation that trade-offs in host resistance to different pathogens have a prime role in maintaining genetic variation for pathogen defence traits.” The previous results from D. melanogaster also refute this expectation and should be cited here.

Response: As noted above, we have revised this sentence to explain that the prevailing expectation is ‘among many evolutionary ecologists’ in Lines 486-7.

Please consider and cite related work on Bt, which is the most studied and widely used biopesticide (used for nearly a century, including decades before transgenic crops). For example, see: Ecol. Ent. 2006, <https://doi.org/10.1111/j.0307-6946.2006.00768.x>
Annu. Rev. Entomol. 2009, <https://doi.org/10.1146/annurev.ento.54.110807.090518>
and J. Econ. Entomol. 2024, <https://doi.org/10.1093/jee/toae077>.

Response: Thank you, we have cited (Janmaat & Myers, 2005), other relevant work in response to another reviewer’s comment (Gassmann et al., 2008), and (Carrière et al., 2010; Tabashnik & Carrière, 2017; Tabashnik et al., 2014) throughout the paper to acknowledge their work on Bt.

Reviewer #2: (No Response)

Reviewer #3: The figure legends were not easy to match to figures as they occured at various intervals in text - this did not help reviewing.

Response: Thank you for highlighting the issue with the placement of figure legends in our manuscript. We have revised the placement of all figure legends so that they directly follow their corresponding mentions in the text. We have also ensured that each figure is referenced within the text at the point where it is most relevant. These changes are intended to facilitate a smoother reviewing and reading experience by making it easier to match figures with their descriptions. We believe these adjustments will significantly improve the manuscript’s clarity and flow for review.

**On the variation in genomic complexity between resistance to chemical control agents and resistance to pathogens:**

Here we present our argument that the genetic control of variation in susceptibility to pathogens tends to involve more loci than are involved in the genetic control of resistance to synthetic insecticides. We acknowledge that there is great variation: not all pathogens are the same, and not all pest responses to selection pressures are the same (we make this point at length in Mangan et al. (2023)). Perhaps one point of ambiguity comes from whether consideration of this issue is motivated by identifying the main loci with major effects on pesticide efficacy, or by a desire to understand the full genomic control of variation in susceptibility to a control agent. It is true that for some control agents there may be one major effect locus that influences resistance, but there may also be other (possibly many other) loci that also influence the resistance trait.

Target-site resistance, of course, generally involves a single gene locus. However, metabolic resistance can involve multiple loci. Referee 1 highlights an excellent recent review by Lu et al. (2024): one important take-home message is that for a sample of 170 cases of resistance (almost all of which are chemical studies) 42% report a monogenic basis.

In the case of pathogens, there are few pathogens used commercially as biopesticides for which there is good information on the genomic basis of resistance: Bt and Cydia pomonella granulosis virus (CpGV) are the exceptions. We disagree with Reviewer 2 that resistance to these two pathogens should be considered monogenic. Although early work on resistance to CpGV did indeed find one gene locus underpinning resistance (Asser-Kaiser et al., 2010), subsequent studies have shown that the situation is more complex (Berling et al., 2013). Indeed the paper that Referee 2 highlights above (Alletti et al., 2017), rather than supporting a simple genetic basis of resistance to this viral pathogen, describes at least three modes of resistance, which apparently have different genomic origins; indeed the introduction to the paper contains the following passage referring to type III resistance to the virus “*Selection and crossing experiments indicated a highly complex polygenic inheritance pattern with some mixed characteristics of type I and type II resistance”.* This clearly underscores the potential for resistance to pathogens to have a complex genetic basis (even for relatively simple viral pathogens). A recent GWAS study suggests that at least eight regions of the genome are involved in resistance to CpGV (Olivares et al., 2023). The case of resistance to Bt, (which Reviewer 2 describes as is “*typically monogenic”*) is interesting because this agent can have a relatively simple mode of action when a single cry toxin is predominantly responsible for pest mortality, meaning single gene changes in the pest can confer considerable resistance. We accept that there are indeed many studies reporting monogenic resistance evolving in the field in crop pests; however, this is not the complete picture. It is worth drawing the distinction between resistance evolved in response to transgenic crops expressing one (or few) Bt insecticidal proteins, and resistance evolved in response to the bacterial pathogen itself (or crops expressing multiple toxins). It is well established that evolution at multiple gene loci can occur to confer resistance (eg various midgut binding receptors); also detoxification pathways can sometimes play a role (Jurat-Fuentes et al 2012). Indeed, there are cases where Bt resistance has been shown to be highly polygenic: such as a recent study finding “*tens of loci*” in *Helicoverpa zea* (Taylor et al., 2024).

In putting forward their argument that pathogen resistance is often monogenic, Reviewer 2 also drew our attention to one study (Briese, 1982) that investigated resistance of potato moths to a virus in the early 1980s. We note that the authors of this paper did find good evidence of a dominant major effect locus; yet they also conclude in their discussion that “*other genes may play a role either by modifying the action of the resistance gene described here or perhaps through other modes of action directly affecting the resistance phenotype. There are several ways in which insects can defend themselves against viruses that could be subject to genetic variability, and hence the overall response of a species to a virus is likely to be polygenic*”. This reflects our viewpoint on the relatively complex genetic basis of resistance to many pathogens. We note that whilst quite a lot of now classic papers have been published on this topic using traditional crossing schemes to investigate Mendelian patterns of inheritance, there are relatively fewer modern GWAS studies to investigate the genetic basis of pathogen resistance in crop pests in high resolution. Nevertheless, there are strong studies in model organisms investigating the genomic basis of pathogen resistance, including to the fungal pathogens we study. We cite some of these in our paper, including Wang et al. (2017) who identified 45 candidate genes associated with resistance to *Metarhizium anisopliae* in *Drosophila melanogaster.*

**References**

Alletti, G. G., Sauer, A. J., Weihrauch, B., Wennmann, J. T., & Jehle, J. A. (2017). Using next generation sequencing to identify and quantify the genetic composition of resistance-breaking commercial isolates of *cydia pomonella* granulovirus. *Viruses*, *9*(9), 250. <https://doi.org/https://www.doi.org/10.3390/v9090250>

Asser-Kaiser, S., Heckel, D. G., & Jehle, J. A. (2010). Sex linkage of CpGV resistance in a heterogeneous field strain of the codling moth *Cydia pomonella* (L.). *J Invertebr Pathol*, *103*(1), 59-64. <https://doi.org/https://www.doi.org/10.1016/j.jip.2009.10.005>

Berling, M., Sauphanor, B., Bonhomme, A., Siegwart, M., & Lopez-Ferber, M. (2013). A single sex-linked dominant gene does not fully explain the codling moth's resistance to granulovirus. *Pest management science*, *69*(11), 1261-1266. <https://doi.org/https://www.doi.org/10.1002/ps.3493>

Briese, D. T. (1982). Genetic basis for resistance to a granulosis virus in the potato moth *Phthorimaea operculella*. *Journal of invertebrate pathology*, *39*(2), 215-218. <https://doi.org/https://doi.org/10.1016/0022-2011(82)90013-1>

Carrière, Y., Crowder, D. W., & Tabashnik, B. E. (2010). Evolutionary ecology of insect adaptation to Bt crops. *Evolutionary applications*, *3*(5-6), 561-573. <https://doi.org/https://www.doi.org/10.1111/j.1752-4571.2010.00129.x>

Cogni, R., Cao, C., Day, J. P., Bridson, C., & Jiggins, F. M. (2016). The genetic architecture of resistance to virus infection in Drosophila. *Mol Ecol*, *25*(20), 5228-5241. <https://doi.org/https://www.doi.org/10.1111/mec.13769>

Ffrench-Constant, R. H. (2013). The molecular genetics of insecticide resistance. *Genetics*, *194*(4), 807-815. <https://doi.org/https://www.doi.org/10.1534/genetics.112.141895>

Gassmann, A. J., Carrière, Y., & Tabashnik, B. E. (2009). Fitness costs of insect resistance to *Bacillus thuringiensis*. *Annual review of entomology*, *54*(Volume 54, 2009), 147-163. <https://doi.org/https://doi.org/10.1146/annurev.ento.54.110807.090518>

Gassmann, A. J., Stock, S. P., Sisterson, M. S., Carrière, Y., & Tabashnik, B. E. (2008). Synergism between entomopathogenic nematodes and *Bacillus thuringiensis* crops: integrating biological control and resistance management. *Journal of Applied Ecology*, *45*(3), 957-966. <https://doi.org/https://doi.org/10.1111/j.1365-2664.2008.01457.x>

Ignoffo, C. M., Huettel, M. D., McIntosh, A. H., Garcia, C., & Wilkening, P. (1985). Genetics of Resistance of Heliothis subflexa (Lepidoptera: Noctuidae) to Baculovirus heliothis1. *Annals of the Entomological Society of America*, *78*(4), 468-473. <https://doi.org/10.1093/aesa/78.4.468>

Janmaat, A. F., & Myers, J. H. (2005). The cost of resistance to *Bacillus thuringiensis* varies with the host plant of *Trichoplusia ni*. *Proceedings of the Royal Society B: Biological Sciences*, *272*(1567), 1031-1038. <https://doi.org/https://www.doi.org/10.1098/rspb.2004.3040>

Kurtak, D., Meyer, R., Ocran, M., Ouédraogo, M., Renaud, P., Sawadogo, R. O., & Télé, B. (1987). Management of insecticide resistance in control of the *Simulium damnosum* complex by the Onchocerciasis Control Programme, West Africa: potential use of negative correlation between organophosphate resistance and pyrethroid susceptibility. *Medical and Veterinary Entomology*, *1*(2), 137-146. <https://doi.org/https://doi.org/10.1111/j.1365-2915.1987.tb00334.x>

Legan, A. W., Allan, C. W., Jensen, Z. N., Degain, B. A., Yang, F., Kerns, D. L., Benowitz, K. M., Fabrick, J. A., Li, X., Carrière, Y., Matzkin, L. M., & Tabashnik, B. E. (2024). Mismatch between lab-generated and field-evolved resistance to transgenic Bt crops in *Helicoverpa zea*. *Proceedings of the national academy of sciences*, *121*(47), e2416091121. <https://doi.org/> <https://doi.org/10.1073/pnas.2416091121>

Lu, X.-P., Xu, L., & Wang, J.-J. (2024). Mode of inheritance for pesticide resistance, importance and prevalence: A review. *Pesticide Biochemistry and Physiology*, *202*, 105964. <https://doi.org/https://doi.org/10.1016/j.pestbp.2024.105964>

Mangan, R., Bussière, L. F., Polanczyk, R. A., & Tinsley, M. C. (2023). Increasing ecological heterogeneity can constrain biopesticide resistance evolution. *Trends in Ecology & Evolution*, *38*(7), 605 - 614. <https://doi.org/> <https://doi.org/10.1016/j.tree.2023.01.012>

Olivares, J., Siegwart, M., Gautier, M., Maugin, S., Gingueneau, L., & Gauffre, B. (2023). Genetic basis of codling moth (*Cydia pomonella*) resistance to the original isolate of C. pomonella Granulovirus (CpGV-M). *Entomologia Generalis*, *43*, 649-658. <https://doi.org/https://www.doi.org/10.1127/entomologia/2023/2052>

Raymond, B., Sayyed, A. H., Hails, R. S., & Wright, D. J. (2007). Exploiting pathogens and their impact on fitness costs to manage the evolution of resistance to *Bacillus thuringiensis*. *Journal of Applied Ecology*, *44*(4), 768-780. <https://doi.org/https://doi.org/10.1111/j.1365-2664.2007.01285.x>

Raymond, B., Wright, D. J., & Bonsall, M. B. (2011). Effects of host plant and genetic background on the fitness costs of resistance to *Bacillus thuringiensis*. *Heredity*, *106*(2), 281-288. <https://doi.org/https://doi.org/10.1038/hdy.2010.65>

Reichelderfer, C. F., & Benton, C. V. (1974). Some genetic aspects of the resistance of Spodoptera frugiperda to a nuclear polyhedrosis virus. *Journal of invertebrate pathology*, *23*(3), 378-382. <https://doi.org/https://doi.org/10.1016/0022-2011(74)90105-0>

Roberts, K. E., Meaden, S., Sharpe, S., Kay, S., Doyle, T., Wilson, D., Bartlett, L. J., Paterson, S., & Boots, M. (2020). Resource quality determines the evolution of resistance and its genetic basis. *Molecular Ecology*, *29*(21), 4128-4142. <https://doi.org/https://doi.org/10.1111/mec.15621>

Shahrestani, P., King, E., Ramezan, R., Phillips, M., Riddle, M., Thornburg, M., Greenspan, Z., Estrella, Y., Garcia, K., Chowdhury, P., Malarat, G., Zhu, M., Rottshaefer, S. M., Wraight, S., Griggs, M., Vandenberg, J., Long, A. D., Clark, A. G., & Lazzaro, B. P. (2021). The molecular architecture of *Drosophila melanogaster* defense against *Beauveria bassiana* explored through evolve and resequence and quantitative trait locus mapping. *G3 (Bethesda)*, *11*(12). <https://doi.org/https://www.doi.org/10.1093/g3journal/jkab324>

Tabashnik, B. E., & Carrière, Y. (2017). Surge in insect resistance to transgenic crops and prospects for sustainability. *Nature Biotechnology*, *35*(10), 926-935. <https://doi.org/https://www.doi.org/10.1038/nbt.3974>

Tabashnik, B. E., Mota-Sanchez, D., Whalon, M. E., Hollingworth, R. M., & Carrière, Y. (2014). Defining terms for proactive management of resistance to Bt crops and pesticides. *Journal of Economic Entomology*, *107*(2), 496-507. <https://doi.org/https://www.doi.org/10.1603/ec13458>

Taylor, K. L., Quackenbush, J., Lamberty, C., Hamby, K. A., & Fritz, M. L. (2024). Polygenic response to selection by transgenic Bt-expressing crops in wild *Helicoverpa zea* and characterization of a major effect locus. *BMC Genomics*, *25*(1), 1247. <https://doi.org/https://www.doi.org/10.1186/s12864-024-11160-x>

Wang, J. B., Lu, H.-L., & St. Leger, R. J. (2017). The genetic basis for variation in resistance to infection in the *Drosophila melanogaster* genetic reference panel. *PLOS Pathogens*, *13*(3), e1006260. <https://doi.org/https://www.doi.org/10.1371/journal.ppat.1006260>
